# Antibiotic Resistant *Enterobacteriaceae* in Milk Alternatives

**DOI:** 10.3390/foods10123070

**Published:** 2021-12-10

**Authors:** Winnie Mukuna, Abdullah Ibn Mafiz, Bharat Pokharel, Aniume Tobenna, Agnes Kilonzo-Nthenge

**Affiliations:** 1 Department of Agriculture and Environmental Sciences, Tennessee State University, 3500 John A. Merritt Boulevard, Nashville, TN 37209, USA; wmukuna@tnstate.edu (W.M.); amafiz@tnstate.edu (A.I.M.); bpokhare@tnstate.edu (B.P.); taniume@tnstate.edu (A.T.); 2 Department of Human Sciences, Tennessee State University, 3500 John A. Merritt Boulevard, Nashville, TN 37209, USA

**Keywords:** multidrug-resistant bacteria, milk alternatives, food safety

## Abstract

The consumption of non-dairy milk is on the rise due to health benefits. Although there is increasing inclination towards milk alternatives (MA), there is limited data on antibiotic resistant bacteria in these substitutes. The aim of this study was to investigate antimicrobial resistance of bacteria isolated from MA. A total of 138 extracts from almonds (*n* = 63), cashew nuts (*n* = 36), and soybeans (*n* = 39) were analyzed for *Enterobacteriaceae*. The identification of the bacteria was based on biochemical and PCR methods. Antibiotic sensitivity was determined by using the Kirby-Bauer disk diffusion technique. Overall, 31% (43 of 138) of extracts were positive for *Enterobacteriaceae.* Ten bacterial species were identified, of which *Enterobacter cloacae* (42.7%) and *Enterobacter cancerogenus* (35.4%) were the most predominant species (*p* < 0.05). Antibiotic resistance was exhibited to vancomycin (88.3%), novobiocin (83.8%), erythromycin (81.1%), which was significantly higher (*p* < 0.05) than in tetracycline (59.5%), cefpodoxime (30.6%), and nalidixic acid (6.3%). There was no resistance displayed to kanamycin and imipenem. ERY-NOV-VAN-TET and ERY-NOV-CEP-VAN-TET were the most common resistant patterns displayed by *Enterobacter cloacae.* The findings of this study suggest that MAs, though considered healthy, may be a reservoir of multidrug resistant opportunist pathogens.

## 1. Introduction

Milk is considered a superior source of micro- and macro-nutrients compared to milk alternatives (MA) [1]. However, its association with increased risks of cardiovascular diseases, diabetes, cancer, and as a principal vehicle for transmission of foodborne pathogens continues to make it unfavorable. Generally, cow milk is frequently consumed and dominates global milk production [2], accounting for 85% of the world’s production, followed by buffalo milk at 11%, goat (2.3%), sheep (1.4%), and camel (0.2%) [3]. However, due to the current changes in lifestyles towards a healthier diet, there has been an increasing trend in the consumption of MA [4]. The U.S. market for MA is increasing and has reached an annual sales volume of $1.8 billion [4]. The increased market growth is attributed to the consumers’ preference for vegan diets, increasing instances of lactose intolerance, and a growing demand for fortified non-dairy food and beverages [5,6,7]. Generally, consumers’ perception is that MA are healthier than milk [8]. Milk alternatives are becoming increasingly popular; however, they are characterized by low protein content, and poor bioavailability of minerals and vitamins [9]. With the increasing demand for these MA, different plants with varying functional attributes are being explored as bases for primary materials for processing [10]. Soymilk, which originated from Asia [11], is the most globally consumed MA while almond milk is the most prevalently used, solely based on sales volume [12]. Other available MA are sourced from cashew nuts, hemp, coconut, rice, etc. [10]. The majority of non-dairy consumers purchase their MA from grocery stores, though a sector of the population make these milk substitutes at home by using raw nuts or seeds. Hence, home-made milk alternative might potentially be contaminated if food safety is not practiced during preparation and storage. Although MA are an intensifying trend, the usage of the term “milk” to mean plant-based substitutes to milk is debatable and is protected by legislation in several countries [8].

There are abundant MA in the market, such as almond, cashew, soy, rice, hazelnut, and oat milk [13,14]. Nuts and seeds, the primary raw materials for milk alternatives, may come into direct contact with soil and be contaminated with pathogenic bacteria at pre- or post-harvest period [15]. It is usually thought that, due to less moisture content, nuts, seeds, and grains are less susceptible to microbial contamination [16]. Regrettably, this attribute does not exempt nuts and seeds from contamination with foodborne pathogens. For instance, *Salmonella* serovar has been detected in almonds, pecans, and peanuts [15,17,18], *E. coli* O157:H7 in walnuts [19], and *Listeria* spp. in peanuts, almonds, cashews, and hazelnuts [20]. Moreover, *Pseudomonas* spp., *Clostridium* spp., and *Klebsiella* spp. have been detected in other nuts [21]. These bacteria and others that are prevalent in raw nuts and seeds belong to the family *Enterobacteriaceae*, the most prevalent human opportunistic pathogens [22].

The increasing frequency of antimicrobial resistant bacteria is a global threat [23]. Accordingly, it is important to study the presence of antimicrobial resistant *Enterobacteriaceae* in MA, especially with the continuous increase trend in consumption. Antimicrobial resistant bacteria cause illnesses that have high morbidity and mortality [24], one of the greatest health challenges in the 21st century [25]. Around 99,000 individuals die every year in the USA owing to drug-resistant infections [26]. Antimicrobial-resistant *Enterobacteriaceae* in milk and milk products has been reported in numerous studies [27]. Just like milk, milk substitutes can also be potential vehicles for transmission of antimicrobial resistant foodborne pathogens to consumers. Antimicrobial resistant pathogens originating from raw nuts or seeds might be transferred to MA during preparation at processing facilities or at home. To the best of our knowledge, there has been limited enquiry of the possible occurrence of antimicrobial resistant bacteria in raw MA. Consequently, this study aims to investigate the presence of opportunist *Enterobacteriaceae* in MA and their resistance to antibiotics used both in human and animal medicine.

## 2. Materials and Methods

### 2.1. Sample Collection and Preparation

**Figure 1:**
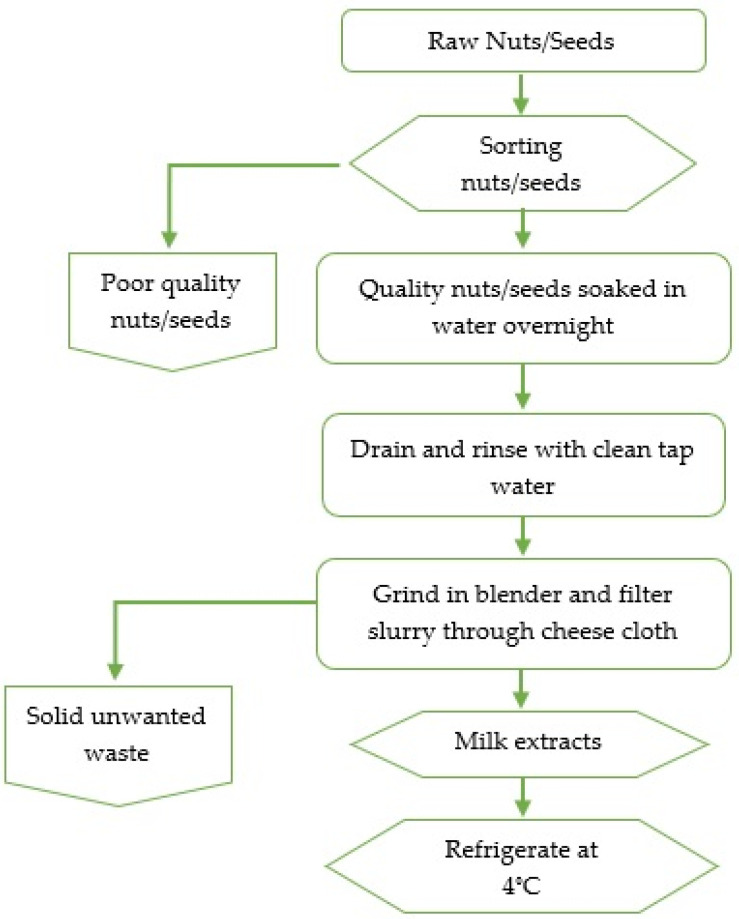
A schematic diagram for extracting milk from almonds, cashews, and soybeans.

Raw nuts (almond, cashew) and soybeans were randomly purchased from 3 local stores in Davidson County, Tennessee, depending on availability. The preparation of almonds, cashew nuts, and soybean extracts involved schematic steps as displayed in the flowchart (Figure 1). Briefly, in duplicates, 5 g of each sample (almonds, cashews, and soybeans) were sorted from unwanted materials (damaged, split seeds or nuts), followed by soaking separately overnight in 45 mL sterile distilled water at room temperature. Next, in duplicates, each sample was disintegrated in a laboratory blender (Waring Division, Dynamics Corporation, New Hartford, CT, USA) for 3 min at high speed. The resulting slurry was filtered through a cheesecloth (Farberware, Fairfield, CA, USA) to attain milk extracts which were then placed in sterile capped containers. A total of 138 extracts (almond nuts = 63, cashew nuts = 36, and soybeans = 39) were analyzed for *Enterobacteriaceae* and AMR by using biochemical and molecular tests.

#### Enrichment of Milk and Bacterial Identification

One ml of nuts and seed extracts was enriched in 9 mL *Enterobacteriaceae* enrichment (EE) broth Mossel enrichment (BD, Sparks, MD) and incubated at 37 °C for 24 h. From each enriched sample, 1µL was streaked onto violet red bile agar (Oxoid, Basingstoke, and Hants, UK) and incubated for 18–24 h at 37 °C. Red to dark purple colonies surrounded by red-purple halos were identified as presumptive *Enterobacteriacea. Enterobacteriacea* isolates and further characterized by using oxidase and API 20E (bioMerieux, Hazelwood, MO, USA) tests. Three colonies per plate were selected for API biochemical testing. Due to the role played by *Klebsiella pneumoniae* and *ronobacter sakazakii* as opportunist pathogens in clinical settings, isolates above the 90% confidence interval were stored at −80 °C for further testing.

### 2.2. DNA Extraction and Confirmation of Klebsiella and Cronobacter Sakazakii

Biochemically identified *K. pneumoniae* and *C. sakazakii* isolates from almond and cashew extracts, respectively, were further confirmed by PCR. DNA was extracted from overnight cultures (≤2 × 10^9^ cells) using the PureLink Genomic DNA Mini Kit (Invitrogen, Carlsbad, CA, USA). DNA concentrations and integrity were determined using a NanoDrop 2000 (Thermo Scientific, Pittsburgh, PA, USA) and agarose gel electrophoresis, respectively. Oligonucleotide primer pairs were synthesized (Operon Technologies, Huntsville, AL, USA) and used to amplify genes of interest. The sequences of the primer pair used for targeting *C. sakazakii* target gene *omp*A (469 bp) was 3′-GGATTTAACCGTGAACTTTTCC-5′ and 5′-CGCCAGCGATGTTAGAAGA-3′ [28,29]. Each reaction mixture (20 μL) contained 4 μL DNA template, 1 μL of each primer (×2), 10 μL master mix, 2 μL RNase free water and, 2 μL coral load (supplied with the kit). *C. muytjensii* (ATCC 51329) was used as a positive control for the detection and identification methods. Reaction conditions for PCR were initial denaturation at 95 °C for 5 min, 30 cycles of denaturation at 95 °C for 30 s, annealing at 55 °C for 1 min, extension at 72 °C for 10 min, and final extension at 72 °C for 10 min.

A Multiplex PCR plus kit (Qiagen, Hillden, Germany) was used to amplify *K. pneumoniae* and *Klebsiella* spp primers in a single reaction [30]. Primer pair 5′-CAA CGG TGT GGT TAC TGA CG-3′ and 5′-TCT ACG AAG TGG CCG TTT TC-3′ targeted gene *rpo*B (108 bp) in *K. pneumoniae* isolates as described by [30], and 5′- CGC GTA CTA TAC GCC ATG AAC GTA-3′ and 5′-ACC GTT GAT CAC TTC GGT CAG G-3′ targeted gene *gyr*A (441bp) in *Klebsiella* spp. [31]. Each 50 μL reaction mixture contained 25 μL of master mix, 5 μL of 10 × primer mix (2.5 μM each primer), 100 ng DNA template, 5 μL Q-solution, 5 μL Coral Load dye, and 10 μL RNase free water. Reaction conditions for PCR were: initial denaturation at 95 °C for 5 min, 35 cycles of denaturation at 95 °C for 30 s, annealing at 60 °C for 90 s, extension at 72 °C for 90 s, and final extension at 68 °C for 10 min. *K. pneumoniae* (ATCC 49131) and *Salmonella typhimuriu*m (ATCC 13311) were used as positive and negative control, respectively. A nexus gradient Thermal Cycler (Eppendorf, Hauppauge, New York) was used for all amplifications. PCR products were electrophoresed in agarose gel stained with 0.1 µg/mL of ethidium bromide (Sigma-Aldrich, Madrid, Spain) and photographed under UV light.

### 2.3. Antibiotic Resistant Profiles

For all identified *Enterobacteriaceae* isolates (*n* = 110), the characterization of the strain resistance/susceptibility profiles was carried out as recommended by the Clinical and Laboratory Standards Institute guidelines [32]. The antimicrobial susceptibility test was conducted on isolates that were identified at ≤90 confidence interval by API 20E system. Antimicrobial disks (*n* = 8), with strength in parentheses were: vancomycin (VAN; 30 μg), novobiocin (NOVO; 30 μg), erythromycin (ERY; 15 μg), tetracycline (TET; 30 μg), cefpodoxime (CEF; 10 μg), kanamycin (KAN; 10 μg), nalidixic acid (NAL; 30 μg), and imipenem (IPM; 30 μg). The results were interpreted as susceptible, intermediate, and resistant based on the Clinical and Laboratory Standards Institute recommendations [32]. *Escherichia coli* ATCC 25922 and *Staphylococcus aureus* ATCC 25923 were used as control strains. Reference standard bacterial strains were verified simultaneously with controls.

### 2.4. Statistical Analysis

The bacterial data were expressed as percentages and analyzed using Microsoft Excel 2016 (Microsoft Corp., Redmond, WA, USA). Chi-square tests were used to measure the significance of difference in the incidence of *Enterobacteriaceae* and antimicrobial resistance. Data were analyzed using SPSS v. 25.0 (IBM SPSS, Chicago, IL, USA). *p* values of less than 0.05 were considered statistically significant.

## 3. Results and Discussion

### 3.1. Enterobacteriaceae in Nuts and Seeds Extract

Overall, 31% (43 of 138) of extracts were positive for *Enterobacteriaceae*. Specifically, *Enterobacteriaceae* isolation rates were 33.3% (21/63), 30.5% (11/36), and 28.2% (11/39) of almond, cashew, and soybean extracts, respectively (data not shown). *Enterobacteriaceae* offers valuable information on the hygienic conditions during food preparation or post-process contamination [33]. Overall, 79.7% (110 out of 138) *Enterobacteriaceae* isolates were identified from almond, cashew, and soybean milk extracts (Table 1).

In 2020, almond milk (MILKLAB and Blue Dimond Almond Breeze Chocolate Almond Milk) recalls were reported in Australia due to contamination with *Pseudomonas* [34]. These recalls support our results that MA can be contaminated with pathogenic bacteria. Our findings also suggest that MA may be contaminated with harmful microorganism. Pathogens such as *Salmonella* serovar., *Listeria* spp., *E. coli* spp., *Campylobacter* spp., *Brucella* spp. or *Shigella* spp. [35,36,37] have been associated with milk.

According to our findings, a total of 10 different commensal and pathogenic genera of *Enterobacteriaceae* were identified with the most common strain being *Enterobacter cloacae* at 42.7% (47 of 110), which was not significantly different (*p* > 0.05) from *Enterobacter cancerogenus* at 35.4% (39 of 110). *E. cloacae* is a commensal microorganism found in human and animal guts and widely found in food, soil, and water [38]. Although *E. cloacae* is not a common foodborne pathogen, its presence in MA is a concern as it is a widely known nosocomial pathogen and the third most prevalent acquired bacteria causing illness in hospital after *E. coli* and *K. pneumoniae* [39]. 

**Figure 2:**
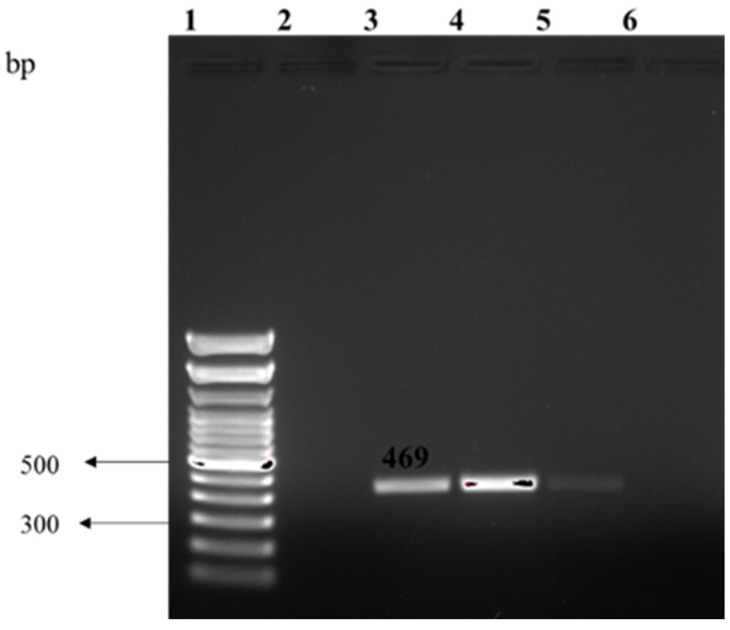
Represents PCR amplification of the *omp*A gene in *Cronobacter sakazakii,* Lane 1: 1 kb ladder; lane 2: negative control; lane 3: positive control; lane 4–5: *C. sakazakii isolates*.

Our results indicate that clinically significant *C. sakazakii* accounted for 2.7% (3 out of 110) of the identified isolates. *C. sakazakii* was only detected in cashew extracts and was confirmed through amplification of the *Omp*A gene (469 bp) (Figure 2).

Earlier findings showed that *Omp*A is a determinant that causes *C. sakazakii* invasion of brain microvascular endothelial cells in vitro, and possibly contributes to pathogenesis of neonatal meningitis [40]. *Cronobacter* spp. is an emerging pathogen and a major concern, especially to hypersensitive clusters of the population including children and the elderly [41,42]. *C. sakazakii* is also considered as an evolving opportunistic pathogen [43] that has been detected in milk, and powdered infant milk among other sources [44]. Although there is no data on the incidence of *C. sakazakii* in MA, nuts and seeds are important raw materials in these substitutes which might be contaminated with pathogenic bacteria at any point during production, harvest, storage, and transportation [45]. At production and harvesting stages, pathogenic bacteria might transfer from the soils onto the nuts/seeds when they are in contact with the ground. One possible scenario is during almond harvesting as was the case of *Salmonella* in almonds grown in California [15]. 

**Figure 3:**
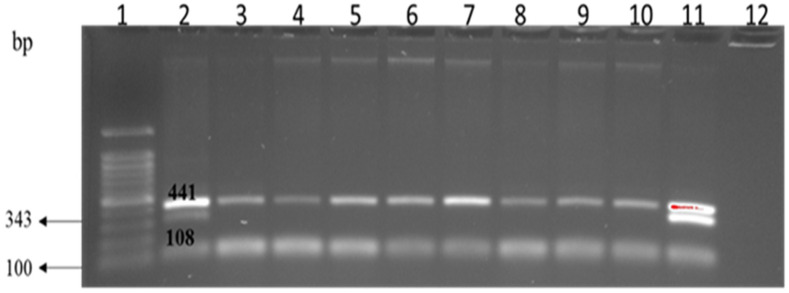
Multiplex PCR amplification of *gyr*A and *rpo*B genes in *K. pneumoniae* and *Klebsiella* spp. Lane 1: 1 kb ladder; lane 2 & 11: positive control; lane 3–10: *K. pneumoniae* and *Klebsiella* spp.; lane 12: negative control.

*K. pneumoniae* spp. *ozaenae* (4.5%) and *K. pneumoniae* spp. *pneumoniae* (2.7%) in the current study were also isolated from almond and cashew extracts, respectively. As these two bacteria are emerging pathogens of concern, they were confirmed by multiplex PCR through amplification of *rpo*B (108 bp) and *gyr*A (441 bp) genes for *K. pneumoniae* and *Klebsiella* spp., respectively (Figure 3).

During harvesting, almond trees are shaken to release the nuts and might stay on the ground for up to 2 weeks before collection [46]. Through this period, bacteria in the soil may be transferred to the hulls which might infiltrate to the kernel as has been demonstrated in *Salmonella* on almonds and pecans [18]. *Klebsiella* spp. have recently become significant pathogens in nosocomial infections [47] such as urinary tract infection, bacteremia, pneumonia, sepsis, and meningitis [48]. With the increased trend in adoption of MA and with some consumers making their nut and seed extracts at home, they may also be at risk of nosocomial infections originating from contaminated and unpasteurized extracts. To avoid potential infections from *Klebsiella* spp., consumers should be encouraged to adhere to food safety practices or drink pasteurized MA.

Other bacteria in the *Enterobacteriaceae* family were also identified in the current study (Table 1). Our findings present *E. vulneris* (1.8%) in soybean extracts. It is possible that the soybeans used in this study were contaminated with *E. vulneris* through soil or water that was used at preharvest or post-harvest. Jain et al. [49] hypothesized that an infant infected with gastroenteritis may have been infected by contaminated formula or water that was used to reconstitute the formula. *Escherichia vulneris* has previously been recovered from water, soil, human beings, and animals [50]. *Rahnella aquatilis* (0.9%) was another *Enterobacteriaceae* isolated from soybean milk extract in our study. *Rahnella aquatilis* is considered a primary and opportunistic pathogen that has been associated with diarrhea and endocarditis [51]. Milk alternatives may be extracted from nuts and seeds that may directly touch the soil during pre- or post-harvest [15]. Hence, restricted precautions must be taken during planting and harvesting of nuts and seeds and processing of MA. Additionally, nuts and seeds should be stored in dry facilities that are protected from rain and ground water, insects and pests, and that have optimal temperatures that avert microbial growth [45]. 

### 3.2. Antimicrobial Drug Resistance in Enterobacteriaceae

Detailed presentation of antimicrobial resistant *Enterobacteriaceae* species from MA extracts is shown in Table 2. In the present study, *Enterobacteriaceae* resistance in isolated bacteria was higher (*p* < 0.05) in vancomycin (90.0%), novobiocin (83.7%), and erythromycin (80.9%) than in tetracycline (60.0%), cefpodoxime (31.8%), and nalidixic acid (6.4%). The majority of *Enterobacteriaceae* in our study are opportunistic pathogens that cause nosocomial infections; hence their antimicrobial resistance might lead to impediments in treating infected individuals [52]. Our findings agree with a previous study that documented *C. sakazakii* resistance to both erythromycin and tetracycline [53]. Occurrence of antibiotic resistant *C. sakazakii* in nut and seed extracts is a concern because antibiotic therapy is a chosen path to avert *Cronobacter* infection in humans [54]. Resistance to erythromycin, tetracycline, vancomycin, and novobiocin was also exhibited by *K. pneumoniae* isolates in nut and seed extracts in our study, hence a concern, since *K. pneumoniae* is a significant multidrug-resistant (MDR) pathogen that causes hospital infections leading to high morbidity and death [55], one of the most severe challenges in clinical practice. 

According to Zhou et al. [56], *Klebsiella*-resistant strains have increased more quickly than those of any other bacteria in the past decade. The consumption of both MA and milk may result in foodborne illnesses if not controlled [57]. According to our study, antibiotic resistant *E. vulneris* was detected in MA. Our data is supported by previous studies [58] which displayed multiple antibiotic resistant *E. coli* strains in milk.

The absence of resistance among all *Enterobacteriaceae* strains to kanamycin was also noted in the current study. Additionally, *Enterobacteriaceae* strains in this study did not display resistance to imipenem which agrees with previous findings [59]. Although no imipenem resistance was indicated in our findings, carbapenems have been used to treat numerous *Enterobacteriaceae* infections, hence there has been a rapid development in their resistance to the same. The rapid spread of carbapenem resistant *Enterobacteriaceae* (CRE) in the community is a national epidemiologic concern, since *Enterobacteriaceae* are common causes of nosocomial and community infections.

A total of seven multidrug-resistance patterns were observed in *Enterobacteriaceae* in this study (Table 3). Out of 110 *Enterobacteriaceae* isolates, 87 (79.1%) from nuts and seed extracts were multidrug-resistant. According to Nguyen et al. [60], an MDR isolate displays resistance to three or more classes of antibiotic. Overall, the most common resistance pattern (ERY-NOV-VAN-TET) in our study was exhibited in by *Citrobacter youngae* (1), *E. cancerogenus* (7), *E. cloacae* (18), *E. vulneris* (1) *Pantoea* spp. 3 (3), and *Rahnella aquatilis* (1) (number of isolates in parenthesis). Forty-four (44) *E. cloacae* and 28 *E. cancerogenus* isolates recovered from nuts and seed extracts were multidrug-resistant (MDR). ERY-NOV-VAN-TET was the most significant (*p* < 0.05) multidrug resistance pattern among *E. cloacae* isolates. *E. cloacae* and *E. cancerogenus* presented a common resistance pattern: ERY-NOV-CEP-NAL-VAN-TET, which was resistant to six out of eight antibiotics.

## 4. Conclusions

Processed MA and milk food safety can be improved by implementation of high sanitary standards that reduce risk of contamination. Milk contamination with micro-organisms can occur before harvest, during milking or postharvest, and in storage. Similarly, MA may be contaminated by use of pathogen tinted nuts or seeds before harvest, during collection, and processing, and in storage. With the increased trend in adoption of MA, consumers may also be at risk of infection with AMR bacteria from ingestion of unpasteurized MA. Therefore, it is imperative that consumers should be educated on safe milk handling practices. Although many consumers are aware of foodborne illnesses, they have limited knowledge of food storage, time, and temperature abuse that may increase bacterial growth.

Although MA are considered healthy, our data suggest that they are reservoirs of antibiotic resistant *Enterobacteriaceae*. Consumers should be aware of the impending risks of ingesting unpasteurized milk substitutes in their homes, which can harbor AMR bacteria that can pose serious health risks.

## Figures and Tables

**Table 1:** Presence (%) of *Enterobacteriaceae* in Nut and Seed Extracts.

Bacterial Species	Total Isolates (N = 110)	No. (%) of ENT Isolates in Extracts		
		Almond Milk (*n* = 56)	Cashew Milk (*n* = 28)	Soy Milk (*n* = 26)
*Enterobacter cancerogenus*	39 (35.4) ^a^	22 (39.28) ^a^	5 (17.9) ^b^	12 (46.2) ^a^
*Enterobacter cloacae*	47 (42.7) ^a^	21 (37.5) ^a^	15 (17.9) ^a^	11 (42.3) ^a^
*Klebsiella pneumoniae* spp. *ozaenae*	5 (4.5) ^bc^	5 (8.9) ^b^	0 (0) ^c^	0 (0) ^c^
*Pantoea* spp. 3	8 (7.3) ^b^	8 (14.2) ^b^	0 (0) ^c^	0 (0) ^c^
*Chryseomonas luteola*	1 (0.9) ^c^	0 (0) ^c^	1 (3.6) ^b,c^	0 (0) ^bc^
*Citrobacter youngae*	1 (0.9) ^c^	0 (0) ^c^	1 (3.6) ^b,c^	0 (0) ^bc^
*Cronobacter sakazakii*	3 (2.7) ^b,c^	0 (0) ^c^	3 (10.7) ^b^	0 (0) ^bc^
*Klebsiella pneumoniae* spp. *pneumoniae*	3 (2.7) ^b,c^	0 (0) ^c^	3 (10.7) ^b^	0 (0) ^bc^
*Escherichia Vulneris*	2 (1.8) ^b,c^	0 (0) ^c^	0 (0) ^c^	2 (7.7) ^c^
*Rahnella aquatilis*	1 (0.9) ^c^	0 (0) ^c^	0 (0) ^c^	1 (3.8) ^c^

N: Total number of *Enterobacteriaceae* isolates. *n*: Total number of *Enterobacteriaceae* isolates from various extracts. ^a–c^ Mean percentages in the same column followed by different letters are significantly different (*p* < 0.05).

**Table 2:** Resistant Antibiotics Profile and *Enterobacteriaceae* Nut and Seed Extracts.

Antibiotics (µg)	No. (%) of *Enterobacteriaceae* Resistant to Antimicrobial Agents									No. (%) of Total Resistant (* R) Isolates
	Almond milk (*n* = 56)			Cashew Milk (*n* = 28)			Soy Milk (*n* = 26)			
	R	I	S	R	I	S	R	I	S	
Erythromycin (15)	48 (85.7) ^b^	8 (14.3) ^b^	0 (0) ^e^	28 (100) ^a^	0 (0)^c^	0 (0) ^d^	13 (50) ^b^	2 (7.7) ^bc^	11 (42.3) ^b^	89 (80.9) ^a^
Novobiocin (30)	51 (91.1) ^b^	1 (1.8) ^d^	4 (7.1) ^d^	27 (96.4) ^a^	13.6) ^a^	0 (0) ^d^	14 (53.9) ^b^	12 (46.1) ^a^	0 (0) ^d^	92 (83.7) ^a^
Cefpodoxime (10)	13 (23.2) ^d^	24 (42.9) ^a^	19 (33.9) ^c^	5 (17.9) ^c^	8 (28.6) ^b^	15 (53.6) ^b^	17 (65.4) ^ab^	5 (19.2) ^b^	4 (15.4) ^c^	35 (31.8) ^c^
NalidixicAcid (30)	5 (8.9) ^e^	2 (3.6) ^cd^	49 (87.5) ^b^	2 (7.1) ^cd^	0 (0) ^c^	26 (92.9) ^a^	0 (0) ^c^	2 (7.7) ^b^	24 (92.3) ^a^	7 (6.4) ^d^
Imipenem (30)	0 (0) ^f^	0 (0) ^d^	56 (100) ^a^	0 (0) ^d^	0 (0) ^c^	28 (100) ^a^	0 (0) ^c^	0 (0) ^c^	26 (100) ^a^	0 (0) ^e^
Kanamycin (10)	0 (0) ^f^	6 (10.7) ^c^	50 (89.3) ^b^	0 (0) ^c^	2 (7.1) ^c^	26 (92.9) ^a^	0 (0) ^c^	2 (7.7) ^bc^	24 (92.3) ^a^	0 (0) ^e^
Vancomycin (30)	56 (100) ^a^	0 (0) ^d^	0 (0) ^e^	28 (100) ^a^	0 (0) ^c^	0 (0) ^d^	15 (57.7) ^ab^	1 (3.8) ^c^	10 (38.5) ^bc^	99 (90.0) ^a^
Tetracycline (30)	31 (55.4) ^c^	17 (30.4) ^a^	8 (14.3) ^d^	14 (50) ^b^	8 (28.6) ^a^	6 (21.4) ^c^	21 (80.8) ^a^	5 (19.2) ^b^	0 (0) ^d^	66 (60.0) ^b^

R = Resistant, I = Intermediate, S = Susceptible (CLSI, 2018). * R = Total number of resistant isolates from all extracts (µg). *n*: Total number of *Enterobacteriacea* isolates from various extracts. ^a–f^ Mean percentages in the same column followed by different letters are significantly different (*p* < 0.05).

**Table 3:** Antibiotic Resistance Patterns of *Enterobacteriaceae* in Nuts and Seed Extracts.

Bacterial Species ^A^	No of Isolates	Resistance Profile ^B^
*Chryseomonas luteola*	1 ^d^	ERY-NOV-CEP-VAN-TET
*Citrobacter youngae*	1 ^d^	ERY-NOV-VAN-TET
*Enterobacter Cancerogenus*	8 ^b,c^	CEP-TET
	1 ^d^	CEP-VAN-TET
	1 ^d^	ERY-CEP-TET
	4 ^c,d^	ERY-NOV-CEP-NAL-VAN-TET
	3 ^c,d^	ERY-NOV-CEP-VAN-TET
	10 ^b^	ERY-NOV-VAN
	7 ^b,c^	ERY-NOV-VAN-TET
	3 ^c,d^	ERY-VAN
	2 ^c,d^	NOV-CEP-TET
*Enterobacter cloacae*	3 ^c,d^	ERY-NOV-CEP-NAL-VAN-TET
	12 ^a,b^	ERY-NOV-CEP-VAN-TET
	11 ^a,b^	ERY-NOV-VAN
	18 ^a^	ERY-NOV-VAN-TET
	2 ^c,d^	NOV-VAN
	1 ^d^	VAN
*Cronobacter sakazakii*	2 ^c,d^	ERY-NOV-VAN
	1 ^d^	ERY-VAN
*Escherichia vulneris*	1 ^d^	ERY-NOV-VAN-TET
	1 ^d^	VAN
*Klebsiella pneumoniae* spp. *ozaenae*	5 ^c,d^	NOV-VAN
*Klebsiella pneumoniae* spp. *pneumoniae*	3 ^c,d^	ERY-NOV-VAN
*Pantoea* spp. 3	3 ^c,d^	ERY-NOV-VAN
	3 ^c,d^	ERY-NOV-VAN-TET
	1 ^d^	ERY-VAN
	1 ^d^	VAN
*Rahnella aquatilis*	1 ^d^	ERY-NOV-VAN-TET

^A^ Bacterial species isolated from milk extracts (MA). ^B^ Antibiotic resistance patterns against eight antibiotics: vancomycin (VAN), novobiocin (NOVO), erythromycin (ERY), tetracycline (TET), cefpodoxime (CEF), kanamycin (KAN), nalidixic acid (NAL), and imipenem (IPM). ^a–d^ Number of isolates in the same column followed by different letters are significantly different (*p* < 0.05).
